# Advances in Foxp3+ regulatory T cells (Foxp3+ Treg) and key factors in digestive malignancies

**DOI:** 10.3389/fimmu.2024.1404974

**Published:** 2024-06-11

**Authors:** Wanyao Wang, Minglu Ding, Qiuhong Wang, Yidan Song, Keyuan Huo, Xiaojie Chen, Zihan Xiang, Lantao Liu

**Affiliations:** ^1^ School of Basic Medicine, Mudanjiang Medical University, Mudanjiang, Heilongjiang, China; ^2^ Mudanjiang Medical University, Mudanjiang, Heilongjiang, China; ^3^ Mudanjiang Hospital for Cardiovascular Diseases, Department of Anesthesiology, Mudanjiang, Heilongjiang, China

**Keywords:** Foxp3, FOXP3+ regulatory T cells, Foxp3 transcriptional and post-translational modifications, digestive system malignancies, immunotherapy targeting Foxp3+Treg

## Abstract

Foxp3+ regulatory T cells (Foxp3+ Treg) play a role in regulating various types of tumors, but uncertainty still exists regarding the exact mechanism underlying Foxp3+ Treg activation in gastrointestinal malignancies. As of now, research has shown that Foxp3+ Treg expression, altered glucose metabolism, or a hypoxic tumor microenvironment all affect Foxp3+ Treg function in the bodies of tumor patients. Furthermore, it has been demonstrated that post-translational modifications are essential for mature Foxp3 to function properly. Additionally, a considerable number of non-coding RNAs (ncRNAs) have been implicated in the activation of the Foxp3 signaling pathway. These mechanisms regulating Foxp3 may one day serve as potential therapeutic targets for gastrointestinal malignancies. This review primarily focuses on the properties and capabilities of Foxp3 and Foxp3+Treg. It emphasizes the advancement of research on the regulatory mechanisms of Foxp3 in different malignant tumors of the digestive system, providing new insights for the exploration of anticancer treatments.

## 1 Introduction

Data from the two most populous countries in the world, China and the United States, indicate that in 2020, China accounted for approximately 23.7% of the global cancer incidence and 30.2% of all cancer deaths, with 4,568,754 new cancer cases and 3,002,899 deaths (1); by 2023, the United States anticipates approximately 1958310 new cancer cases and 609820 cancer deaths (2). Thus, Medical professionals have shown that Foxp3+Treg expression is elevated in high-grade digestive malignant tumors, such as liver, pancreatic, and stomach cancers, and is typically linked to a poor prognosis for patients. This discovery has been made when investigating the formation mechanism of diverse malignant tumors (3–6).

This article mainly summarizes the research progress on the expression mechanism and function of Foxp3+Treg and Foxp3 related features and functions in digestive system malignant tumors, which have been publicly published in publications such as Pubmed. We also discuss the application of immunotherapy techniques to inhibit the tumor immunosuppressive effect of Foxp3+Treg based on the regulation of Foxp3+Treg expression in the tumor microenvironment, thereby improving the patient’s own immune and anti-tumor ability, and providing new ideas for personalized treatment of tumor patients.

## 2 Foxp3+ regulatory T cells (Foxp3+ Treg) and their key factors

### 2.1 Foxp3+Treg

Foxp3+ Treg cells predominantly secrete Foxp3, a subset of T cells that regulate the body’s immune system by exerting immunosuppressive effects. Foxp3+ Treg can be acquired in two ways: the first is through maturation in the thymus and release into the periphery, at which point Foxp3+ Treg are also known as natural regulatory T cells (nTregs); another method of production is through Transforming Growth Factor-β (TGF-β) Induced regulatory T cells (iTreg). These cells protect their own reactive lymphocytes from immunological reactions, thereby maintaining their immunity and tissue stability (7) (**Table 1**).

**Table 1:** Changes in the expression of Foxp3 and Foxp3+ T cells.

Level	Type of regulation	Factors of action	Mechanim of action	Cancer type/cells	References
**Transcription**	Foxp3 mRNA and Protein down	c-Rel	c-Rel acetylation negatively regulates Foxp3 promoter activation	EL4 and MT-2 cells	(8)
	Foxp3 mRNA and Protein down	Enolase -1	Enolase-1 is specifically recruited to the Foxp3 promoter and its CNS2 regulatory region		(9)
	Foxp3 mRNA and Protein up	SAMHD1	Phosphorylation of the T592 site in the TCR/CDK2/SAMHD1 pathway interacts directly with Foxp3 to inhibit dNTPase activity and enhance the stability of Foxp3 mRNA	human peripheral blood	(10)
	Foxp3 mRNA and Protein And Foxp3+Treg Up	Tet2	TGF-β/Uhrf1/Tet2 enables it to act on the CpG island of Foxp3 CNS2, enhancing the stability of Foxp3 mRNA and thus maintaining the stability of Foxp3+Treg.		(11)
	Foxp3 mRNA and Protein down	flicr	flicr acts in cis on Foxp3 transcriptional regulation and reduces Foxp3 stability	C57BL/6J mice	(12)
	Foxp3 mRNA and Protein down	PI3K	PI3K/AKT/mTOR pathway activation reduces Foxp3 production		(13)
	Foxp3 mRNA and Protein down	miR-125b	miR-125b inhibits Foxp3 mRNA and protein expression by targeting the 30 UTR of Foxp3	Thyroid cancer	(14)
	Foxp3 mRNA and Protein Up	miR-4281	miR-4281 directly binds to the human Foxp3 promoter and upregulates Foxp3 protein expression		(15)
**Translation and post-translation modifications**	Protein of Foxp3 up The function of Foxp3+Treg Up	PRMT 1	Methylation of arginine residues 48 and 51 on Foxp3 by PRMT 1		(16)
	Foxp3 Protein down The function of Foxp3+Treg down	PARP-1	PARP-1 promotes Stub1-mediated Foxp3 degradation and polyubiquitination	HEK293T cells	(17)
	Foxp3 Protein up	USP7	Up-regulation of USP7 reduces ubiquitination of Foxp3 and stabilizes Foxp3 protein expression	HCT116 (p53WT) and human prostate cancer PC-3 (p53MUT) cell	(18)
	Foxp3 Protein up	USP44	USP44 activation removes ubiquitination modifications from K3-linked ubiquitin chain linkages and stabilizes Foxp3 protein	BALB/c mice	(19)
	Foxp3 Protein down	Phosphorylation of Tyr-191	Phosphorylation of the tyrosine residue Tyr-191 of Foxp3	MCF-7 cells	(20)
	Foxp3 Protein up	Phosphorylation site Ser418	The presence of the phosphorylation site Ser418 at the C-terminus of Foxp3 promotes the protein expression of Foxp3		(21)
	Foxp3 Protein up	HAT p300	HAT p300 mediates Foxp3 acetylation modifications competing for co-interacting ubiquitination modifications and inhibits degradation of Foxp3	HEK293 cells	(22, 23)
	Foxp3 Protein down	SIRT1	SIRT1 negatively regulates the stability of Foxp3 by deacetylating the Foxp3 lysine acetylation site (K31, K262 and K267).	293T and Jurkat cells	(23, 24)
**Other** **Protein level**	The function of Foxp3+Treg up	HIF-1α	Binding of HIF-1α to Foxp3 protein disassembles its ubiquitination	Seven human lung cancer cell lines (H358, H460, H524, H1650, H838, H1975 and A549)	(25)
	The function of Foxp3+Treg up Foxp3 Protein up	STAT3	The STAT3/Foxp3 axis in ESCC tissues promotes Foxp3 expression through STAT3 modification of the Foxp3+Treg transcription process	ESCC	(26)
	The function of Foxp3+Treg up	COX-2	IL-33/ST2/Foxp3+Treg/COX-2/PGs regulatory pathway that promotes COX-2 secretion by Foxp3+Treg	PDAC	(27)
	Foxp3+Treg up	CCL5	Recruitment of Treg to the tumor microenvironment by CCL5 in concert with Foxp3	PDAC	(28)
	Foxp3 Protein up	AKT1	AKT1/FOXP3/CerS6 axis	PDAC	(29)
	The function of Foxp3+Treg up	FoxM1	FoxM1 was accompanied by a significant increase in the number of Foxp3+Treg	GC	(30)
	Foxp3 Protein down	miR-34a	MKL1/miR34a/FOXP3 axis represses FOXP3 expression via miR-34a	GC	(31)
	Foxp3 Protein down	miR-133a-3	Binding of miR-133a-3p to the untranslated region 3'-UTR at the 3' end of Foxp3 inhibits	GC	(32)
	The function of Foxp3+Treg up	Helios	Co-expression of Foxp3 with Helios enhances the inhibitory profile of Treg more than each molecule acting alone	CRC	(33)
	The function of Foxp3+Treg up	TCF-1	Decrease in TCF-1 increases Foxp3 binding to downstream gene regulatory elements, which in turn promotes Treg expression	CRC	(34)
	Foxp3+Treg up	HDGF	PolyIC/TLR/HDGF axis activation promotes Foxp3+Treg expression	HCC	(35)

### 2.2 Foxp3

The transcription factor Foxp3 belongs to the multi-domain Fox family of forkhead box proteins. Proline and other amino acids make up the majority of the N-terminal region, which binds to different protein molecules to carry out transcriptional inhibition functions. For example, it can inhibit the production of NF-AT-mediated transcription-activating factor IL-2 (36, 37); the stable C-terminal forked head domain (FKH) can recognize and bind specific DNA sequences. Central zinc finger transcriptional regulation and oligomerization modification with leucine domain (38). Foxp3 is primarily produced by T cells; however, non-Treg cells can also generate some Foxp3. Foxp3, a crucial transcription factor in regulatory T cells (Treg cells), regulates various functions and processes, such as development and maturation. The function of Treg cells may be inhibited by a lack of or low expression. Significantly, human X-linked and autoimmune diseases, including immune system disorders, several endocrine disorders, intestinal diseases, X-linked syndrome (IPEX syndrome), and others, can result from the functional loss or mutation of the Foxp3 gene (39, 40). The primary features include various autoimmune disorders affecting organs, significant allergies, and excessive inflammation. T cell differentiation and function may be influenced by the interactions of Foxp3 with other factors. Through its antagonistic effects on Toll-like receptors (TLRs), Foxp3 contributes to the reprogramming of T cell metabolism, enhancing T cell oxidative phosphorylation (OXPHOS) and fatty acid oxidation capacity. Additionally, Foxp3 collaborates with the original glycolytic pathway to produce energy, ensuring that Treg cells have the essential resources for proliferation and preventing cell apoptosis (41, 42). Conversely, Foxp3 suppresses the glycolysis and Myc signaling pathways when it binds to the oncogene Myc promoter, which impacts T cell metabolism (43).

**Figure 1:**
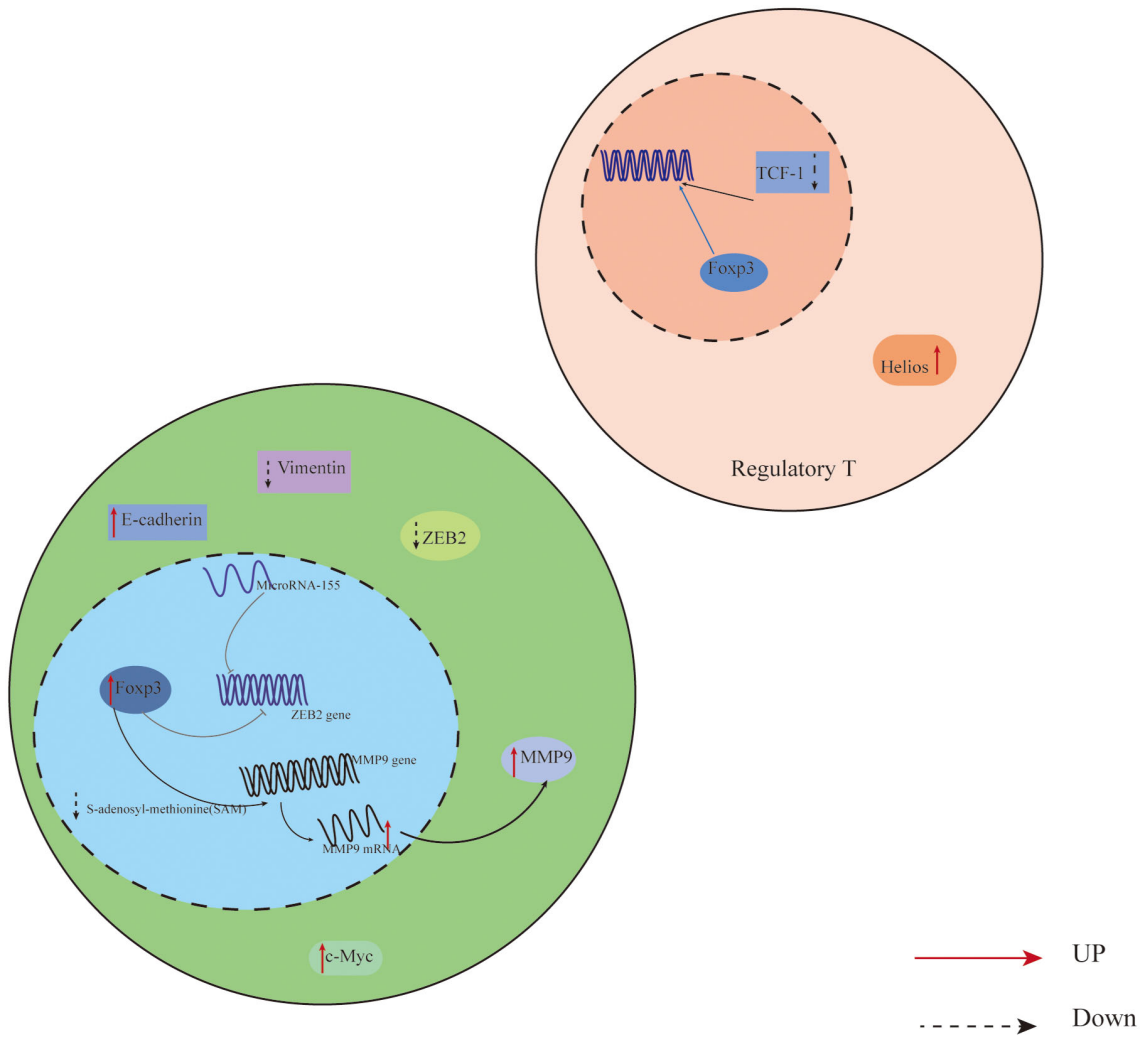
Expression of Foxp3 in relation to T cells and cancer cells in the CRC tumor microenvironment. In colon cancer cells, Foxp3 and microRNA-155 work together to synergistically regulate the transcription of Zinc finger E-box binding homology box 2 (ZEB2), which suppresses ZEB2 expression and boosts anti-tumor immunity, reducing cancer cell growth and metastasis; In addition, Overexpression of Foxp3 also promotes MMP9 expression through the SAM cycle; The decrease of TCF-1 leads to an increase in the binding of Foxp3 to downstream gene regulatory elements, thereby promoting the expression of Treg, inhibiting the proliferation of effector T cells, and promoting the progression of CRC. ZEB2,the Zinc finger E-box binding homology box 2; MMP9, Matrix Metallo Protein 9; TCF-1,T Cell Transcription Factor 1.

Additionally, Foxp3 can control the body’s immunity by directly or indirectly influencing downstream components in tumor cells. In their investigation of non-small cell lung cancer cells, Peng et al. (44) discovered that Foxp3 overexpression in the tumor microenvironment can suppress anti-tumor immunity and promote the proliferation of cancer cells. Direct binding of Foxp3 to the LINC00885 promoter can upregulate the production of proteins associated with the epithelial mesenchymal transition (EMT), thereby promoting the growth and invasion of cervical cancer cells (45). Furthermore, numerous clinical investigations have established that overexpression of Foxp3 is associated with a poor prognosis and low survival rate in cancer patients (46, 47). For instance, Foxp3 expression is higher and survival is lower in individuals with oral squamous cell carcinoma (48). On the other hand, patients with specific malignancies have been shown to benefit from elevated Foxp3 expression. According to published research, patients with high levels of VEGF and CD44 expression in breast cancer have a comparatively short survival period. Additionally, there is a negative correlation between Foxp3 expression and VEGF and CD44 expression. On one hand, Foxp3 directly inhibits the activity of the VEGF promoter through a specific forkhead binding motif, which leads to the inhibition of angiogenesis in breast cancer by suppressing the expression of VEGF, consequently downregulating VEGF (49); On the other hand, Foxp3 binds to the promoter of CD44 coding gene to suppress breast cancer metastasis, thereby hindering the progress of breast cancer (50). In addition, Gal-1 regulates the anti-tumor properties of Foxp3 by binding with the FKH domain of Foxp3 in Foxp3-positive breast cancer cells, thereby maintaining the stability of cancer cells (51). According to reports, Foxp3 is a downstream target of p53 mediated cell aging, promoting the aging of epithelial cancer cells by inducing the expression and generation of p21 and ROS (52). It Collaborates with microRNA-155 to regulate the transcription process of Zinc finger E-box binding homology box 2 (ZEB2), inhibiting the expression of ZEB2 in colon cancer. This inhibition leads to the suppression of cancer cell proliferation and metastasis, enhancing anti-tumor immunity (53) (**Figure 1**). Therefore, due to the widespread expression of Foxp3 in tumor cells, it has a dual role in tumor induced proliferation or inhibition. Studying the regulatory mechanisms of Foxp3+ Treg cells and Foxp3 involvement in malignant tumors is of great significance for understanding and developing treatments for each disease.

Expression of Foxp3 in relation to T cells and cancer cells in the CRC tumor microenvironment. In colon cancer cells, Foxp3 and microRNA-155 work together to synergistically regulate the transcription of Zinc finger E-box binding homology box 2 (ZEB2), which suppresses ZEB2 expression and boosts anti-tumor immunity, reducing cancer cell growth and metastasis; In addition, Overexpression of Foxp3 also promotes MMP9 expression through the SAM cycle; The decrease of TCF-1 leads to an increase in the binding of Foxp3 to downstream gene regulatory elements, thereby promoting the expression of Treg, inhibiting the proliferation of effector T cells, and promoting the progression of CRC. ZEB2,the Zinc finger E-box binding homology box 2; MMP9,Matrix Metallo Protein 9; TCF-1,T Cell Transcription Factor 1.

## 3 Foxp3’s expression regulation mechanism

**Figure 2:**
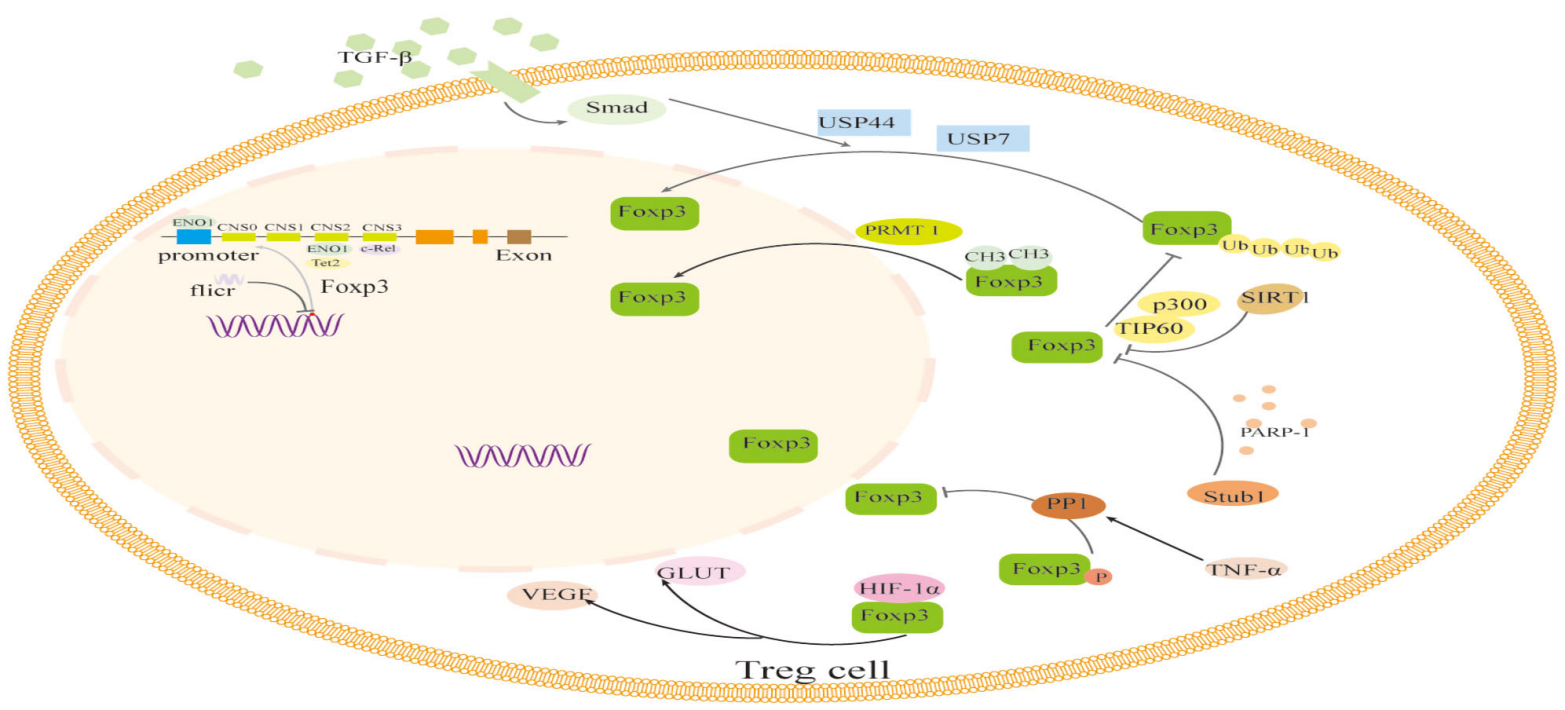
The regulation of transcription and translation modifications related to Foxp3 in Foxp3+ Treg cells. The transcription of Foxp3 and the activation process of Foxp3 are regulated by binding to non-coding sequences such as c-Rel, ENO1, and Tet2, like CNS0-3, during transcription. In addition, the long non-coding RNA (lncRNA) Fricr is similar to the Foxp3 genome and can act in cis on the transcription process of Foxp3. Foxp3's post-translational regulation process mainly involves the methylation of PRMT1, glycosylation of PARP-1, cooperative ubiquitination of USP44 and USP7, dephosphorylation of PP1, acetylation regulation of SIRT1 and p300, and glucose metabolism process. The interaction of HIF-1α with Foxp3 protein and other factors can influence the expression and function of Foxp3. TGF-β, Transforming Growth Factor β; USP44, Ubiquitin Specific Peptidase 44; USP7, Ubiquitin Specific Peptidase 7; PRMT1, Protein arginine methyltransferase 1; PARP-1, Poly (adp-ribose) polymerase 1; PPI, Phosphoproteinase 1; TNF-α, Tumor necrosis factor-α; GLUT, The glucose transporter; VEGF, Vascular Endothelial Growth Factor; HIF-1α, Hypoxia-inducible factor-1α; Tet2, Tet methylcytosine dioxygenase 2.

The translated Foxp3 protein precursor is altered by methylation, acetylation and deacetylation, phosphorylation, ubiquitination and deubiquitination, glycosylation, and other modifications in addition to epigenetic modifications that control Foxp3 expression during transcription. Additionally, Foxp3 expression may be impacted directly or indirectly by changes in sugar metabolism or short RNAs (**Figure 2**).

The regulation of transcription and translation modifications related to Foxp3 in Foxp3+ Treg cells. The transcription of Foxp3 and the activation process of Foxp3 are regulated by binding to non-coding sequences such as c-Rel, ENO1, and Tet2, like CNS0–3, during transcription. In addition, the long non-coding RNA (lncRNA) Fricr is similar to the Foxp3 genome and can act in cis on the transcription process of Foxp3. Foxp3’s post-translational regulation process mainly involves the methylation of PRMT1, glycosylation of PARP-1, cooperative ubiquitination of USP44 and USP7, dephosphorylation of PP1, acetylation regulation of SIRT1 and p300, and glucose metabolism process. The interaction of HIF-1α with Foxp3 protein and other factors can influence the expression and function of Foxp3. TGF-β, Transforming Growth Factor β; USP44, Ubiquitin Specific Peptidase 44; USP7, Ubiquitin Specific Peptidase 7; PRMT1, Protein arginine methyltransferase 1; PARP-1, Poly (adp-ribose) polymerase 1; PPI,Phosphoproteinase 1; TNF-α, Tumor necrosis factor-α; GLUT, The glucose transporter; VEGF, Vascular Endothelial Growth Factor; HIF-1α, Hypoxia-inducible factor-1α; Tet2, Tet methylcytosine dioxygenase 2.

### 3.1 Transcriptional and post-transcriptional regulation

It has been discovered that the conserved non-coding sequence CNS0–3 includes the Cage1 site, which is situated 1500 bp upstream of the core promoter, and the Cage2 site, which is situated 2000 bp upstream, as well as other required regulatory regions for Foxp3 gene expression (54). The TCR on T cells surface activates Foxp3 transcription negatively by binding to NF-κB. The conserved CNS3 regulatory region of Foxp3 is then joined with the c-Rel of the family. Conversely, acetylated c-Rel on the site inhibits Foxp3 production by adversely regulating the activation of the Foxp3 promoter (8). Alternatively, Foxp3 expression can be inhibited by the specific recruitment of enolase-1 in the glycolytic pathway to the Foxp3 promoter and its CNS2 regulatory region; however, this study also has shown that TCR activation leads to an increase in the cell’s ability to uptake glucose and glycolysis, which, in turn, leads to an increase in the expression of Foxp3 (9). Reports state that long non-coding RNA (lncRNA) fricr is comparable to the Foxp3 genome and has the ability to act cis on Foxp3’s transcription process, decreasing its stability (12); additionally, miR-125b has been shown in thyroid cancer research to target Foxp3 and suppress its mRNA and protein production while increasing cancer cells’ susceptibility to cisplatin treatments (14). Reduced Foxp3 expression is advantageous for enhancing the therapeutic benefit of patients on anti-cancer medications, as miR-4281 directly binds to the Foxp3 promoter, upregulates the expression of Foxp3 protein, and promotes the immunosuppressive role of Foxp3+Treg (15). To better guide anti-cancer treatment, it is imperative to have a thorough understanding of the mechanisms behind the activity of non-coding RNAs (ncRNAs), as they are also crucial in regulating the Foxp3 process.

The TCR signaling pathway has a distinct regulatory mechanism that manifests a twofold regulatory effect throughout the transcriptional activation process of Foxp3. Deoxyribonucleoside triphosphate triphosphate hydrolase (SAMHD1) co-localizes with ODNps25 in the cytoplasm and interacts directly with Foxp3 through phosphorylation of T592 in the TCR/CDK2/SAMHD1 pathway, which increases the stability of *Foxp3* mRNA by inhibiting the activity of the dNTPase (10). The DNA demethylating Tet2 enzyme (a member of the TETase family) is increased by high-intensity TCR signaling stimulation (55) or TGF–induced phosphorylation of Uhrf1, which enables it to act on the CpG island of Foxp3 CNS2 and preserves the stability of the Foxp3+ Treg (11). However, Stephan Sauer et al. (13) has shown that stimulation of the PI3K/AKT/mTOR pathway reduces Foxp3 production and persistent TCR signaling prevents naive CD4+T cells from differentiating into Foxp3+CD4+ T cells. In summary, further research is necessary to fully understand the effects of Foxp3 involvement on cancer cells and Foxp3+Treg function in particular tumor microenvironments, as well as to demonstrate the advantageous role of Foxp3 therapeutic blockade in future immunotherapy strategies, which will offer fresh concepts for clinical targeted therapy. This is even though we have clarified the specific regulatory mechanisms of some Foxp3 transcription processes in different tumor types.

### 3.2 Post-translational regulation

#### 3.2.1 Methylation and glycosylation

Foxp3+Treg**’**s inhibitory activity is improved by protein arginine methyltransferase 1 (PRMT 1), according to research by Yuki Kagoya and colleagues (16). The adp-ribosylation enzyme Poly (adp-ribose) polymerase 1 (PARP-1) enhances the multimerization (adp-ribosylation) of Foxp3 in contrast. Foxp3 is made unstable by PARP-1 through Stub1-mediated degradation and polyubiquitination, which inhibits Foxp3+Treg**’**s inhibitory effectand strengthens the immune system (17).

#### 3.2.2 Ubiquitination and deubiquitination

Foxp3+Treg regulation depends on ubiquitination and deubiquitination. It is discovered that activating or up-regulating the deubiquitinating enzyme USP7 in Treg cells could decrease Foxp3 ubiquitination, stabilize Foxp3 protein expression, and enhance Foxp3+Treg’s immunosuppressive effect. Conversely, inhibitors that prevent USP7 from being deubiquitinated can reduce Foxp3+Treg’s ability to suppress the immune system, which can allow tumor cells to evade the immune system (18). Furthermore, TGF-β, as The Smad signaling pathway, activates ubiquitin specific peptidase 44 (USP44), which can stabilize Foxp3 protein and eliminate ubiquitination modifications of K3 linked ubiquitin chain connections; the combined expression of USP44 and USP7 stabilizes Foxp3 expression, and the absence of USP44 in Treg cells can promote anti-tumor immunity and impede tumor growth (19).

#### 3.2.3 Phosphorylation

The expression of associated proteins like S-phase kinase associated protein 2 (Skp2), matrix metallo protein 9 (MMP9), and vascular endothelial growth factor-A (VEGF-A) is inhibited by the tyrosine kinase LCK-dependent phosphorylation of Foxp3, particularly at tyrosine residue Tyr-191 (20). Additionally, it has been demonstrated that the Ser418 phosphorylation site, which is located at the C-terminus of Foxp3, is phosphorylated in autoimmune diseases. This phosphorylation site can promote the expression of Foxp3 proteins and preserves the inhibitory function of Foxp3+ Treg cells. In contrast, when the Ser418 site is specifically dephosphorylated by TNF-alpha-activated phosphoproteinase 1 (PP1), it can inhibit the function of Foxp3+ Treg cells (21). UBC9 is the only E2 enzyme for Small Ubiquitin-like Modifier (SUMO), which can also target the UBC9 promoter region to up-regulate SUMOization to regulate Foxp3+ Treg cell function after phosphorylation modification of the Y342F site of Foxp3 in human breast cancer cells (56).

#### 3.2.4 Acetylation and deacetylation

Foxp3 protein acetylation, a mechanism that prevents proteasomal degradation, also regulates the level of Foxp3 expression. Loosdregt (22) reported for the first time that acetylation directly controled the level of Foxp3 protein. High levels of Foxp3 acetylation may prevent the protein from being polyubiquitylated, which raises the level of Foxp3 protein and aids in the development of T cells into Foxp3+ Treg. The acetylation modification of Foxp3 by histone acetyltransferases (HAT) p300 can compete with the ubiquitination modification co-acting on lysine residues and restrict Foxp3 degradation; on the other hand, the deacetylation modification of Foxp3 by histone acetyltransferase SIRT1 can obstruct Foxp3 synthesis. Additionally, the Foxp3 protein can be stabilized and acetylated by the TIP-p300-Foxp3 complex, which is formed when TIP60 and p300 work together (23). Additionally, Hye-Sook Koon (24) recently have discovered that SIRT1 modifies Foxp3**’**s lysine acetylation sites (K31, K262, and K267) by deacetylation in order to adversely control Foxp3 stability.

### 3.3 Altered glucose metabolism

According to the study’s findings, Foxp3+Treg expression increased in the majority of tumors, and while the tumor microenvironment was anaerobic, HIF-1 aggregation was present (57). In this situation, HIF-1can bind to the Foxp3 protein and can inhibit HIF-1 degradation, and at the same time, Foxp3 can increase HIF-1’s downstream target genes, including VEGF and the glucose transporter protein (GLUT) (58). Lung cancer cell growth is aided by the MIF/NF-ƘB/HIF-1 pathway of macrophage migration inhibitory factor, which keeps the Warburg effect-related factors stable (25). In conditions with high lactate and low sugar, oxidizing lactate to pyruvate facilitates nicotinamide adenine dinucleotide (NAD) regeneration and oxidative phosphorylation. Foxp3+Treg benefits from changes in tumor glycolysis because it strengthens its immunosuppressive capacity in the tumor microenvironment and encourages tumor cell survival (59). Nonetheless, other researchers have also noted that Foxp3 protein’s ubiquitination is disrupted when HIF-1 α binds to it, which in turn impairs T cell development and, ultimately, the production and functionality of Foxp3+Treg (60).

In conclusion, changes in glucose metabolism enable tumor cells to maintain their energy source, promote growth, and adapt better to hypoxic environments. Still, there are additional elements in the glucose metabolism pathway that are regulated by Foxp3 and HIF-1α. We must conduct more research to better understand the connection between Foxp3 and its function.

## 4 Mechanisms of Foxp3 and Foxp3+ Treg cell pro-cancer in malignant tumors of the digestive system

### 4.1 Esophageal cancer

Esophageal Carcinoma (ESCC) is a cancerous tumor that primarily affects the esophageal epithelium. According to research, the upstream regulatory factor p53 of miR-149–3p (61) is inhibited from being ubiquitinated when miR-5b-1p binds to the molecular sponge rcRUNX3 (62) or lncRNA MEG3. This helps to upregulate Foxp3 expression in ESCC. By increasing Foxp3 expression through STAT3 modification of Foxp3+Treg transcription process, activating the STAT3/Foxp3 signaling pathway within ESCC tissue hinders macrophage phagocytosis and increases Foxp3+Treg immunosuppressive activity, which helps ESCC evade the body’s immune response (26). Furthermore, upon recognition of its receptor ST2, IL-33 attracts a significant number of Foxp3+Tregs to congregate in the stroma of ESCC, thereby initiating the IL-33/ST2/Foxp3+Treg/COX-2/PGs regulatory pathway, encouraging Foxp3+Treg secretion of cyclooxygenase-2 (COX-2) and enabling the conversion and production of prostaglandins (PGs). These actions work in concert to preserve the immune system’s stability and suppress the tumor microenvironment’s effect, encouraging the proliferation and growth of ESCC cells (27).

### 4.2 Pancreatic Cancer

The most prevalent type of pancreatic cancer is pancreatic ductal adenocarcinoma (PDAC), and an increase in Foxp3 in the tumor microenvironment of pancreatic cancer affects immune cells (DCs), which are antigen-presenting cells, impairing the body’s ability to mount an immune defense (63). It has been shown that Foxp3 works synergistically to control the recruitment of chemokine CCL5 to Treg cells in the tumor microenvironment to promote the immune escape of PDAC. Foxp3 also directly interacts with the promoter region of programmed cell death-ligand (PD-L1) to promote PD-L1 inhibition of effector T cell activation (3, 28, 64). Foxp3 also confers a suppressive phenotype on Tregs in pancreatic cancer cells by repressing the transcriptional activation of several T cell-stimulated target genes, such as Interleukin-2 (IL-2) (65). It is hypothesized that CTLA-4 could be linked to Foxp3+ T cell suppression of effector T cell function and mediate PDAC immune escape because it is another cytotoxic T lymphocyte-associated antigen with immunosuppressive effects that is expressed more frequently (66). The tissues of pancreatic tumors have elevated expression of the Ceramide synthase (CerSs) isoform CERS6. AKT1-mediated phosphorylation of Foxp3 at the S418 site aids in maintaining the production of CERS6, and elevated levels of CERS6 mRNA predict a worse prognosis for PDAC patients, according to ex vivo research. In addition, CERS6 promotes the p53 mutation that causes PDAC. As a result, inhibiting the AKT1/FOXP3/CERS6 axis can be a possible tactic to prevent the growth of pancreatic tumors (29).

### 4.3 Gastric cancer

Genetic variations and epigenetic changes are linked to an increased risk of gastric cancer (GC) (67). In GC tumor tissues, Foxp3 protein is expressed more intracellularly (68). When compared to GC tumor tissues, the CD8/Foxp3 ratio in GC paracancerous tissues is much greater, and the elevated expression of PD-L1 could signal a bad prognosis for GC patients (69). The number of Foxp3 + Treg cells is significantly higher than that of paraneoplastic tissues, and the release of COX-2 by Foxp3 + Treg leads to the induction of Prostaglandin E2 (PGE2) expression. These factors together inhibits the anti-cancer effects of effector T cells (6), allowing GC to avoid immune attack and promoting its immune escape and proliferation. Additionally, using COX-2 inhibitors to treat GC offers the chance to reduce Foxp3+ Treg activity. Foxp3 interacts to the PSMD7 promoter and stimulates PSMD7 expression, both of which are up-regulated in gastric cancer tissues, boosting GC cell proliferation and blocking apoptosis (70). With extensive research, Li et al. (30) discovered that FoxM1, along with an increase in the number of Foxp3+Treg, increased significantly in gastric cancer tissues, that overexpression of FoxM1 and Foxp3+Treg favored GC infiltration and invasion, and that inhibition of the regulatory pathways of FoxM1 and Foxp3 could block GC proliferation, implying that the combination of FoxM1 and Foxp3+Treg could be used as a biomarker for diagnosis and prognostic survival of clinical gastric cancer patients. Foxp3+Treg cells share the functional characteristics of Foxp3+Treg cells and RORt-expressing TH17 cells, respectively, for having enhanced immunosuppressive and pro-inflammatory effects, while inhibiting the production of anticancer factors, such as IFN-γ, granzyme B, and other anticancer factors by effector T cells, resulting in an imbalance in the body’s immunity to achieve anticancer immune escape, which is detrimental to the prognosis of patients (71).

Furthermore, investigations have demonstrated that Foxp3 plays an oncogenic function in GC. Foxp3 promotes p21 protein expression by binding to the p21 promoter region, which inhibits cancer cell proliferation; however, in the presence of a large amount of inflammatory infiltration in the GC, Foxp3 has an enhanced interaction with p65, which reduces binding to the p21 promoter, thus contributing to cancer cell proliferation (72); S-phase kinase-associated protein 2 (SKP2) is associated with a double negative feedback loop of p21 and p27, which can lead to the hypothesis that overexpression of Foxp3 in GC inhibits the oncogenic effects of SKP2 (73); the MKL1/miR34a/Foxp3 pathway inhibits the expression of Foxp3 and promotes the proliferation of GC (31). Foxp3 inhibits GC cell proliferation by activating the apoptotic signaling pathway, and increasing Foxp3 expression can increase the expression of pro-apoptotic genes such as PARP, caspase-3, and Casp9, effectively inducing apoptosis in GC cells, and vice versa (74). Foxp3 suppression or binding to miR-133a-3p can limit Foxp3 expression, which stimulates GC cell proliferation and autophagy, helping to deplete and remove damaged cells and maintain cancer cell homeostasis (32). Meanwhile, Foxp3 reduces COX2 expression and cell metastasis as a negative regulator of NF-ƘB activity, presenting new therapeutic and diagnostic alternatives for gastric cancer (75, 76).

### 4.4 Colorectal cancer

The study of CRC markers and their associated signaling pathways may offer suggestions for targeted therapy treatment of colon cancer because colorectal cancer (CRC) is a common cancer of the digestive system, the incidence rate is rising annually in China, and the survival rate of patients is poor (77). Treg cells have different subtypes, and Treg accumulates at the tumor site. On the one hand, it may be due to the co expression of chemokines and other products such as Foxp3 and Helios produced by tumor cells and stroma in the TME of CRC, which can enhance the inhibitory characteristics of Treg more strongly than other molecules acting alone. In addition, The expression levels of PD-1/CTLA-4 and PD-1/CD39 in the subgroup of Treg are elevated. They have a synergistic effect in inhibiting T cell activation and function, as well as inhibiting tumor specific immune responses, thereby helping tumor cells evade T cell immune attacks and promoting cancer cell progression (33). On the other hand, in the presence of TGF - β, it can also drive the expression of tumor infiltrating Treg cells. In addition, The Treg density of FOXP3 in tumor tissue is higher than that in normal colon mucosa, and its increased expression is associated with poor CRC survival rate. Overexpression of Foxp3 promotes MMP9 expression through the SAM cycle, promoting liver metastasis in CRC (78–82) (**Figure 1**). TCF-1 and Foxp3 can bind to regulate the same genes. TCF-1 can control T cell development in the thymus (34). As TCF-1 levels drop in CRC, more Foxp3 can bind to downstream gene regulatory elements, promoting the development of Treg, inhibiting the growth of effector T cells, and advancing CRC (83) (**Figure 1**). While Foxp3+Treg is not positively correlated with Foxp3+ cancer cells, and purely high expression of Foxp3+Treg has no correlation with CRC prognosis, some researchers have discovered, contrary to the majority of findings, that high expression of Foxp3 in Foxp3+ cancer cells is correlated with poor prognosis (84). Additionally, Liu et al.’s research (85) demonstrated that Foxp3 expression was markedly down-regulated in colon cancer stem cells, that activating the Foxp3/NF-κB/COX2 pathway could prevent COX2’s transcriptional activation and impede the proliferation of colon cancer stem cells, and that Foxp3 was able to inhibit the growth of cancer cells. In summary, the research on Foxp3 expression in CRC is valuable and has predictive implications, but further investigation is required to comprehend its role in different cell types. This knowledge will be beneficial for improving CRC treatment and prevention strategies.

### 4.5 Hepatocellular carcinoma

Hepatocellular carcinoma (HCC) refers to malignant tumors of the liver, including primary and metastatic hepatocellular carcinoma, and HCC patients are often detected in peripheral mononuclear lymphocytes with a significant increase in the expression of the factors Foxp3 and RORt, which are closely related to the development of HCC, compared to normal cells (86, 87). Yong Huang (88) has discovered that in HBV-infected people, IL-17-producing Th17 cells congregate with Foxp3+ Treg cells, facilitating HCC progression. MMP12 might promote Foxp3+Treg infiltration in tumor tissue; TLR4 might obliquely attract Foxp3+Treg to the tumor location by interacting with TGF-β and macrophages (89, 90). Through promoting Treg cell polarization, it facilitates HCC immunological escape. High basal levels of lnc-EGFR specifically bind to EGFR in HCC patients, blocking its interaction and ubiquitination with c-CBL, stabilizing it and enhancing its own and downstream activation of the AP-1/NF-AT1 axis. This prolongs the lifespan of EGFR, drives Treg differentiation, stifles CTL activity, and encourages the growth of HCC (91). Furthermore, it was discovered that TLR ligands, particularly polyIC (a synthetic double-stranded RNA polyinosinic polycytidylic acid), stimulated an increased capacity of hepatocellular carcinoma cell line (CCL-9.1) in mice to release Hepatoma Derived Growth Factor (HDGF), as well as Foxp3+ Treg cells to proliferate, which together inhibit the release of perforin and granzyme B from effector CD8+ T cells into the tumor microenvironment for the purpose of assisting cancer cell immune escape (35). The binding of the lncRNA NEAT1 to the Foxp3 binding site results in high expression of its downstream target gene, pyruvate kinase PKM2, and over-expression of NEAT1/Foxp3 promotes PKM2 transcriptional activation (92), which promotes HCC proliferation by enhancing the aerobic glycolysis pathway.

Furthermore, Foxp3 are found to have an oncogenic influence in both HCC and CRC. Mutations in the FKH structural domain of Foxp3 has been found at the transcriptional level in HCC tumor tissues, affecting the function of controlling the expression of target genes (93). Foxp3 expression is down-regulated in HCC tissues, but P62 expression is up-regulated. Low Foxp3 expression and P62 over-expression are found to be closely connected to a decrease in overall survival in HCC patients (94). Liu et al. (95) has discovered that the Foxp3 promoter and CpG region are hypermethylated by NA (cytosine-5)-methyl transferase 1 (DNMT1), which can limit Foxp3+Treg function and negatively controls HCC progression. Moreover, Gong et al. (96) also can confirme that Foxp3 exhibits oncogenic effects in HCC through *in vitro* and *in vivo* experiments, and Foxp3 regulates the TGF-β/smad3/4 pathway to recognize and directly or indirectly act on the Myc promoter region of oncogenes to inhibit oncogene expression; at the same time, the over-expression of Foxp3 could promote the increase of apoptotic marker Bax and expression of apoptosis inhibitor p53, which promote cancer cell apoptosis (97, 98).

## 5 The mechanism of action of Foxp3 and Foxp3+Treg cells in other malignant tumors

Regulating T cell infiltration is a major obstacle to immunotherapy in TME and is often associated with poor prognosis. We found that the accumulation of Foxp3+Treg cells in lung cancer is higher than that in normal tissues, and the increased infiltration of regulatory T cells into the core tumor area may be an independent predictor of poor overall survival in non-small cell lung cancer (NSCLC) patients (99). We found *in vitro* culture that over-expression of Foxp3 in Treg cells enhances the activity and invasiveness of related immune cells, indicating that an increase in Foxp3 levels in the tumor microenvironment may promote tumor cell growth (44); And the correlation between TGF - β and Foxp3 was also shown in the lungs of non-small cell lung cancer patients; Compared with patients with limited period NSCLC, the incidence of circulating CD4+CD25+Foxp3+Treg cells in advanced NSCLC patients is significantly increased, and the frequency of circulating CD4+CD25+Foxp3+Treg cells is negatively correlated with interleukin (IL) -17 and positively correlated with serum IL-10 levels (100). Therefore, an increase in circulating CD4+CD25+Foxp3+Treg cells may be involved in the pathogenesis of NSCLC. In addition, analysis of 70 cervical cancer patients found that, The expression of Foxp3 and VISTA is associated with clinical staging, The group with double positive expression of Foxp3 and VISTA had the worst prognosis, The positive expression of Foxp3 and VISTA may serve as independent prognostic factors for cervical cancer, providing strong evidence for immunotherapy of cervical cancer (101). However, in breast cancer patients, the expression of chemokine receptor CCR4 in tumor infiltrating Tregs is higher than that in peripheral Tregs, The expression of FOXP3 in breast cancer tissue is higher than that in normal tissue, and overexpression of FOXP3 is associated with better prognosis. Knockdown FOXP3 with siRNA *in vitro* can promote the migration and invasion of human breast cancer MCF-7 cells; In addition, CCL22 and CCL17 released by tumor cells and tumor associated macrophages can attract CCR4+Tregs to the tumor site, and FOXP3 and HAT1 can together epigenetically alter the promoter on CCR4+Tregs, providing space for FOXP3 binding and CCR4 gene activation. Overexpression of FOXP3 increases the infiltration of CCR4+Tregs cells, leading to reduced anti-tumor immune response and tumor progression. These findings indicate that, as a transcriptional activator of CCR4 and a regulator of Treg invasion, FOXP3 overexpression is related to the good prognosis of breast cancer and plays an important role in the tumor microenvironment of breast cancer (102–104).

Based on the above research, we found that Foxp3+Treg cells are widely involved in the immune regulation of different types of tumor cells, but their roles vary in different types of tumors, with both positive and negative effects. Therefore, we need to further explore and analyze the specific functions of Foxp3+Treg cells in a certain tumor, in order to provide ideas for the treatment of tumors.

## 6 Foxp3+Treg cells in immune treatment for tumors

Tumor immunotherapy is progressively being used in clinical practice to treat a variety of malignant tumors, in addition to conventional surgical resection, radiation, and chemotherapy (105–107). This has a significant positive impact on the clinical outcomes of various cancers and gives cancer patients hope. Tumor immunotherapy is the use of novel medications to actively or passively interact with intracellular signaling pathways and receptors linked to the transformation and progression of cancer. This results in an immune response specific to the tumor, inhibits the growth and survival of cancer cells, and kills or inhibits tumors (108). To enhance the body’s anti-tumor response and increase the effectiveness of cancer patients’ treatments, tumor immunotherapy mostly consists of immune checkpoint inhibitors (ICIs), adoptive cell transfer therapy (ACT), anti-tumor monoclonal antibodies (mAbs), tumor vaccines, small molecule antibodies, etc (109–111).

Patients with various cancers showed an increase in Foxp3+Treg cells in their tumor microenvironment. These cells can help tumor cells evade the immune system by blocking anti-tumor immunity. This is typically linked to a patient’s clinical prognosis and poor tumor development (3, 112–115). Since Foxp3 is the primary distinctive marker of Foxp3+Treg cells (3), Foxp3-targeting medications include enzyme inhibitors, both synthetic and natural; Foxp3’s research and development typically entail changing process-related enzymes, downstream small molecules, or Foxp3 itself in order to interfere with its transcription and translation processes, limit its output and lowers the expression of the immunosuppressive subgroup Foxp3+Treg of CD4+T cells as well as their ability to fight tumors (116). Foxp3-microRNA or Foxp3-shRNA generated by R&D design can be delivered to tumor cells in an experimental study to decrease Foxp3 expression and Foxp3+ Treg function using Ultrasound-Targeted Microbubble Destruction (UTMD) for gene delivery (117). The synthetic peptide can inhibit Foxp3 protein-protein (PPI) interactions, and as a result, it has the potential to be employed as a novel medication to disrupt Foxp3+Treg function. Hawley et al. (118) investigated that hydrocarbon-anastomosing α-helix (SAH) peptide could target the Foxp3 homodimer protein region, blocking interactions between Foxps, disrupting the signaling pathway, and decreasing Foxp3-mediated immunosuppression. However, the peptide cannot have membrane-penetrating properties and needs to be transfected into the cells, but the specific mechanism of action is still unclear and needs to be explored in depth. In light of the fact that Foxp3 also occurs in the organism as a complex and that Foxp3 collaborates with Rcor1/2 to create the COREST complex, inhibitors of the COREST complex block Rcor1 endoenzyme activity, which in turn prevents Foxp3+Treg activity (119). Methotrexate (MTX), a medication used to treat psoriasis, controls immunological homeostasis by boosting Foxp3 mRNA expression and Treg cell expression (120). To enhance the body’s ability to fight tumors, it is crucial to thoroughly evaluate strategies for blocking the Foxp3 regulatory pathway, considering both the cancer-promoting and cancer-fighting effects of Foxp3 in tumors.

Moreover, a group of antibodies called immune checkpoint inhibitors (ICIs) work by blocking inhibitory receptors on immune cells. In medical settings, these immunotherapeutic drugs target immune cells to some extent and show significant anti-cancer effects in cancer patients. Currently, immune checkpoint blockers (ICBs) can directly target Foxp3+Treg by focusing on co-inhibitory receptors like anti-CTLA4, anti-PD-1, and anti-PD-ligand 1 (PD-L1). This approach can enhance anti-tumor immune responses by hindering the immunosuppressive function of Foxp3+Treg. However, despite the considerable success, a considerable number of patients have not shown positive responses (121). However, the loss of Treg homeostasis can also result in potentially fatal autoimmune adverse events (irAEs) because Tregs play a critical role in immunological tolerance maintenance and the prevention of autoimmune disorders (122) (**Table 2**). The most frequent and early adverse event (irAE) is skin toxicity, and immune regulatory effects triggered by ICIs and targeted therapy are required for anti-tumor effectiveness. But because cancerous cells and healthy skin mucosal tissue share a signaling route, immunotherapy-induced stimulation may also impact healthy skin tissue, which can result in the development of autoimmune skin disorders (123–127). According to preliminary observations, the therapy of ICIs was also observed to have an impact on the cardiovascular system, including conditions such as myocarditis, fulminant myocarditis, arrhythmia, venous thromboembolic illness, acceleration of atherosclerosis, atherosclerosis, and other associated cardiovascular issues (128, 129). Treatment with inhibitors of inflammatory response modifiers (ICIs) has been linked to an increase in cardiovascular events not just during the initial few weeks of treatment but even months or years after treatment commencement; However, the risks of cardiac toxicity vary throughout combination immunotherapy techniques and ICI treatment regimens. When using a single ICI treatment, CTLA-4 may be more cardiacally toxic than PD-1 or PD-L1. When using dual therapy, there is a greater chance of cardiac toxicity with dual ICI therapy than with single chemotherapy or single targeted therapy (130). Moreover, a lot of people have grown resistant to medications over time (131–134). The resistance mechanisms of sporadic MSI-H endoplasmic reticulum cells were revealed to be antigen processing, presentation flaws, and induction disorders in interferon response in a prospective phase 2 pilot study based on pembrolizumab in patients with recurrent MSI-H endometrial cancer. Patients with high microsatellite instability (MSI-H)/mismatch repair defects (dMMR) appear to be able to slow their progression with surgical resection or local therapy, in addition to ongoing pembrolizumab discontinuation studies (145). Furthermore, ICIs and targeted therapy can cause rheumatoid arthritis clinical symptoms, nail toxicity, oral mucosal toxicity, and hair loss (135–140, 146–148). In addition, ICI treatment can also lead to hepatitis (141–144). Eleonora et al. evaluated the features of liver injury using ICIs and discovered that liver biopsy is useful in determining the diagnosis and degree of liver injury in patients with metastatic cancer undergoing immune-mediated hepatitis. According to this study, patient-centered care is crucial and could eventually prevent the need for needless systemic corticosteroid therapy (149). Patients receiving targeted and immune checkpoint inhibitor (ICI) therapy for multiple health conditions need to carefully follow the Society for Immunotherapy of Cancer’s (SITC) Clinical Practice Guidelines for Adverse Events Related to Immune Checkpoint Inhibitors and the National Comprehensive Cancer Network’s (NCCN) updated guideline for managing immune-related adverse events (IRAEs). It is crucial for patients to work closely with dermatologists and other specialists, and make informed decisions about their medications based on scientific guidance (150–152). This approach helps minimize the impact of treatment side effects on patients’ well-being and encourages their commitment to and enthusiasm for the therapy.

**Table 2:** The loss of Treg homeostasis can also lead to life-threatening autoimmune adverse events (irAEs).

IrAEs (types)	Specific manifestations or symptoms	References
Skin toxicity	Systemic lupus erythematosus, vitiligo, acne like rash, non-specific papules, can also induce eczematous like lesions or psoriasis lesions, lichen like dermatitis, dry skin syndrome, and itching	(123–127)
Cardiovascular system and cardiotoxicity	Cardiomyositis, fulminant myocarditis, arrhythmia, arrhythmia, venous thromboembolic disease, accelerated atherosclerosis, atherosclerosis, hypotension and other related cardiovascular problems	(128–130)
Drug resistance	The use of immune checkpoint inhibitor therapy has expanded, leading to secondary resistance and immune escape resulting in no response to the inhibitor	(131–134)
alopecia	Appearing non scar hair loss similar to alopecia areata (AA), eosinophilic folliculitis	
Oral mucosal toxicity	The main symptoms of oral toxicity include infection, facial neuropathy (such as sensory disorders), taste disorders, dry mouth syndrome, decreased salivary gland function, jawbone necrosis, and oral toxicity mainly manifested as mucosal lesions, decreased salivary gland function, or facial neuropathy	(135–138)
Nail toxicity	Nail detachment, clubbing, paronychia, nail peeling, nail brittleness, and slowed nail growth rate	(135, 139, 140)
Hepatotoxicity	hepatitis	(141–144)

Maintaining a balanced immune response is a significant challenge when targeting and suppressing Tregs within the tumor microenvironment (TME) of cancer patients, without affecting overall self-tolerance. To address this, modifying nanobiomaterials to create intelligent nanocarriers for drug delivery systems (DDS) can be helpful. By delivering ICI to specific targets such as Foxp3+Treg, the immunosuppressive TME can be altered to boost anti-tumor immunity, while minimizing side effects and improving the effectiveness of ICI treatment. This approach leads to safe and efficient cancer immunotherapy, with these specially designed nanoparticles offering advantages over traditional delivery methods (153). Thus, more study is needed in the direction of targeting tumor-specific Tregs without compromising Treg homeostasis overall and preventing the development of irAEs. Furthermore, because of the comprehensive study of tumor immunology theory and the ongoing advancements in technology, ICIs in conjunction with other anti-cancer therapeutic modalities have been approved for use in a variety of cancer types (154). For example, research on triple negative breast cancer (TNBC) suggests that combining immunotherapy with cancer vaccines and immune checkpoint inhibitors may be beneficial for non-immunogenic tumors. In a study by Liu et al., nanoparticles (NPs) were created to deliver mRNA vaccines encoding the tumor antigen MUC1 to dendritic cells (DCs) in lymph nodes, aiming to activate tumor-specific T lymphocytes. By combining the mRNA vaccine with an anti-CTLA-4 monoclonal antibody, the anti-tumor effects can be maximized. NP-based mRNA vaccines that target mannose receptors on DCs have shown *in vivo* efficacy in expressing tumor antigens and inducing a strong cytotoxic T lymphocyte response in TNBC 4T1 cells. Furthermore, combining vaccines with anti-CTLA-4 monoclonal antibodies has been found to significantly enhance the anti-tumor immune response compared to using either treatment alone. These results highlight the potential synergistic benefits of NP-based mRNA and CTLA-4 inhibitors in treating TNBC, with NPs serving as a carrier for mRNA vaccine delivery (155). Thus, it is anticipated that the combined use of other tumor treatment techniques will lead to new advancements in cancer eradication.

## 7 Conclusion

Based on the above discussion, we should now have a better understanding of the function of Foxp3+Treg in tumor immunity by reviewing a wide range of prior studies. Particularly, the investigation of the mechanism regulating the expression of its key characteristic transcription factor Foxp3 in digestive malignancies offers a broad array of supportive therapeutic targets and early diagnostic markers with predictive value for tumor diagnosis and treatment. Nevertheless, little is known about how Foxp3, a crucial regulatory component in tumor immunology, affects the immune response that relies on Foxp3+Treg cells in the tumor microenvironment. From the standpoint of Foxp3, we still do not fully understand how Foxp3 is expressed during the post-translational modification stage or the precise mechanisms underlying its function in various cell types. Thus, more studies on Foxp3 are required to enhance our understanding of Foxp3+Treg cells and to guide the development of new immunotherapy approaches. Furthermore, specific pharmacological technologies should be developed for treatment because the Foxp3 pathway is currently underutilized in the development of anti-tumor therapies, as indicated by available experimental evidence. By disrupting signaling pathways, regulating the immune balance of the tumor system, mediating Foxp3 expression and the immunosuppressive effect of Foxp3+ Treg cells, and employing a variety of treatment modalities simultaneously to enhance anti-cancer immunity, we aim to achieve the goal of curative treatment for tumors.

## Author contributions

WW: Writing – review & editing, Writing – original draft, Visualization, Investigation. MD: Writing – review & editing, Supervision, Investigation. QW: Writing – review & editing, Investigation. YS: Writing – review & editing, Investigation. KH: Writing – review & editing, Investigation. XC: Writing – review & editing, Investigation. ZX: Writing – review & editing, Investigation. LL: Writing – review & editing, Supervision, Resources, Funding acquisition.
